# Attacking Postoperative Metastases using Perioperative Oncolytic Viruses and Viral Vaccines

**DOI:** 10.3389/fonc.2014.00217

**Published:** 2014-08-12

**Authors:** Lee-Hwa Tai, Rebecca Auer

**Affiliations:** ^1^Centre for Innovative Cancer Research, Ottawa Hospital Research Institute, Ottawa, ON, Canada; ^2^Department of Surgery, University of Ottawa, Ottawa, ON, Canada

**Keywords:** metastasis, postoperative period, oncolytic viruses, viral vaccines, cancer, perioperative immunostimulation, natural killer cells, surgical stress

## Abstract

Surgical resection of solid primary malignancies is a mainstay of therapy for cancer patients. Despite being the most effective treatment for these tumors, cancer surgery has been associated with impaired metastatic clearance due to immunosuppression. In preclinical surgery models and human cancer patients, we and others have demonstrated a profound suppression of both natural killer (NK) and T cell function in the postoperative period and this plays a major role in the enhanced development of metastases following surgery. Oncolytic viruses (OV) were originally designed to selectively infect and replicate in tumors, with the primary objective of directly lysing cancer cells. It is becoming increasingly clear, however, that OV infection results in a profound inflammatory reaction within the tumor, initiating innate and adaptive immune responses against it that is critical for its therapeutic benefit. This anti-tumor immunity appears to be mediated predominantly by NK and cytotoxic T cells. In preclinical models, we found that preoperative OV prevents postoperative NK cell dysfunction and attenuates tumor dissemination. Due to theoretical safety concerns of administering live virus prior to surgery in cancer patients, we characterized safe, attenuated versions of OV, and viral vaccines that could stimulate NK cells and reduce metastases when administered in the perioperative period. In cancer patients, we observed that *in vivo* infusion with oncolytic vaccinia virus and *ex vivo* stimulation with viral vaccines promote NK cell activation. These preclinical studies provide a novel and clinically relevant setting for OV therapy. Our challenge is to identify safe and promising OV therapies that will activate NK and T cells in the perioperative period preventing the establishment of micrometastatic disease in cancer patients.

## Surgical Stress Promotes the Formation of Metastases

Surgical resection is the mainstay of therapy for most solid malignancies but, even with complete resection, many patients harbor microscopic residual disease and ultimately die of a recurrence (1). Our group (2, 3) and others have clearly demonstrated, using different animal and tumor models, that surgery promotes the formation of metastatic disease (4–11) and the number of metastatic deposits is directly proportional to the magnitude of surgical stress (6, 12). In clinical studies, a complicated postoperative course correlates with inferior cancer survival and increased incidence of metastases (13, 14). A number of perioperative changes have been proposed to explain the promotion of metastases formation following surgery including (1) dissemination of tumor cells during the surgical procedure (15–20), (2) local and systemic release of growth factors, such as vascular endothelial growth factor (VEGF) (21, 22), and (3) cellular immune suppression. The cellular immune suppression following major surgery appears to peak at 3 days (23) following surgery but may persist for weeks (7, 23–25). It is hypothesized to be mediated by secretion of stress hormones, such as glucocorticoids (26, 27), catecholamines (27–29), and prostaglandins (26). It is characterized by both plasma cytokine changes [a decrease in IL-2 (30), IL-12 (31) and an increase IL-6 (27, 30, 32, 33), IL-10 (34)] and a decrease in the number and function of circulating lymphocytes [cytotoxic T cells (35), dendritic cells (DC) (36) and natural killer (NK) cells (2, 3, 37)].

The postoperative stress response represents a diverse set of physiological changes that have evolved to ensure that the host can heal following major tissue trauma. These changes, however, involve pathways and mediators that can be exploited by cancer cells to facilitate metastatic spread. While a number of correlative studies have demonstrated an association between some of these changes and the enhanced formation of metastases following surgery, few mechanistic studies have been undertaken to understand it. This review will focus on the importance of both innate and adaptive postoperative cellular immune suppression, specifically NK and cytotoxic T cell postoperative dysfunction and make the case for the use of preoperative oncolytic viruses (OV) and viral vaccines to prevent the promotion of cancer metastases following surgery.

## Surgical Stress Inhibits NK Cell Function and Antigen-Specific CD8^+^ T Cell Function

Both the innate and adaptive immune system play a significant role in anti-tumor immunity. As integral members of the innate immune system, NK cells are involved in the direct killing of cells displaying abnormalities linked to infection, malignancy, or transplantation (38, 39). Immunosurveillance of the host by NK cells for malignant cells results in direct cytotoxicity and the production of cytokines to enhance the immune response (39).

Natural killer cell dysfunction following surgery, as measured in a standard [51-Cr]-release assay, has been documented in both human patients (3, 25, 40–42) and animal models (3, 5, 7, 40, 41, 43). Postoperative NK cell suppression correlates with increased metastases in animal models of spontaneous (3, 9) and implanted (3, 10, 11) metastases, while in human studies low NK activity during the perioperative period is associated with a higher rate of cancer recurrence and mortality in a number of different cancer types (44–46). Despite the large number of studies that have documented postoperative NK cell dysfunction, very few studies have thoroughly characterized and directly explored the mechanism of this suppression (9, 11, 47). Our laboratory has clearly defined a role for NK cells in the development of postoperative metastases (2). Using several reproducible mouse models of surgical stress, including B16 melanoma, CT26 colon cancer and 4T1 breast cancer, our laboratory has demonstrated a consistent and significant (two- to fourfold) increase in the formation of experimental and spontaneous pulmonary metastases following surgery. In these experimental models, surgery markedly reduced NK cell total numbers in the spleen and affected NK cell migration. Further, *ex vivo* and *in vivo* tumor cell killing by NK cells were significantly reduced in surgically stressed mice. To establish that NK cells play the crucial mediating role in clearing tumor metastases following surgery, we transferred surgically stressed NK cells into NK-deficient mice (IL-2γR-knock out) and observed enhanced lung metastases in tumor-bearing mice compared to mice who received untreated NK cells (3). Transfer of NK cells labeled with the NK specific marker DX5 from surgically stressed and no surgery control donors into naive recipient mice represents the first *in vivo* evidence that links surgery to the spread of cancers via NK cells (3). In human studies, we have also confirmed that postoperative cancer surgery patients had markedly reduced NK cell cytotoxicity (3).

The adaptive immune system and more specifically CD8^+^ T cells responses have received the majority of the attention from the cancer immunity field. Of recent interest in our lab is the impact of surgical stress on the development and maintenance of an acquired T cell-mediated anti-tumor immune response. A global reduction in T cell numbers and function post-surgery has been documented in preclinical studies and cancer patients (35). However, the effects of tumor-associated antigen (TAA)-specific T cells have not been evaluated and represent a current focus of research interest in our lab.

## Postoperative Cellular Immune Suppression is Reversible

Fortunately postoperative immune suppression is reversible, so while the postoperative period provides a window of opportunity for cancer cells to metastasize and grow, it also provides a window of opportunity to intervene, by supporting or further stimulating the immune system, and, in doing so, attenuate the development of cancer recurrences (48, 49). Based on promising preclinical results (8, 50, 51), clinical trials of preoperative non-specific immune stimulation with low-dose recombinant IFNα (52) or IL-2 (53–58) have demonstrated less NK and T cell suppression following surgery. In two randomized studies of patients undergoing resection of colorectal cancer (CRC) primary tumors (58) and hepatic metastases (57), preoperative low-dose subcutaneous (s.c.) IL-2 was associated with an improved prognosis. In the first study, 86 CRC patients with stage II or III disease were randomized to receive low-dose IL-2 twice a day for 3 consecutive days prior to surgery or no preoperative treatment. At a median follow-up of 54 months, there were significantly few recurrences in the IL-2 group (21.4 vs. 43.1%, *p* = 0.03) and a trend toward improved overall survival (OS). In the second study, 50 CRC patients with Stage IV disease, undergoing curative or palliative surgery, were randomized to the same two treatment arms. The median progression-free survival (PFS) and OS were significantly longer in the preoperative IL-2 group. While these studies were not designed to evaluate cancer outcomes, a Phase II trial in 120 patients undergoing resection for renal-cell carcinoma has demonstrated a significant improvement in 5-year PFS with preoperative IL-2 (74 vs. 62%, *p* = 0.02) (54). Moreover, in all of these studies, preoperative IL-2 was safe and well tolerated with adverse events limited to pyrexia (Grade I–III). A few other non-conventional immunomodulators have been evaluated for their ability to boost cellular immunity in the perioperative period including cimetidine (59, 60), mistletoe extract (61, 62), and granulocyte colony-stimulating factor (GMCSF) (63). Despite the paucity, the data are promising and perioperative treatment strategies, aimed at stimulating the cellular immune system warrants further study. As outlined in the remainder of this review, OV are an attractive agent to reverse perioperative immune suppression.

## Why Use Perioperative Oncolytic Viruses for Immune Stimulation? A Multipronged Approach for a Multifactorial Problem

Oncolytic viruses are not considered a “traditional” immunotherapy but their multiple mechanisms of action provide several advantages over traditional cytokine immune stimulants in the complex postoperative period. First, the immune stimulation provided by an OV is a more “physiological” immune stimulus, engaging and maturing DC, which in turn activates NK and T cells. The multitude of cytokines and chemokines, stimulate the appropriate picomolar concentration, by a systemic virus infection would be impossible to replicate even with the most carefully designed cytokine cocktail. Second, the OV will selectively replicate in and kill residual cancer cells, providing a direct cytolytic effect to remaining micrometastases, but also delivering the immune response to the tumor selectively. Finally, there is strong rationale to hypothesize that OV could infect and replicate better in the postoperative state because of the surge of growth factors such as VEGF, providing a therapeutic advantage for OV in postoperative cancer patients.

## Preclinical Evidence for NK Cell Activation with Perioperative Oncolytic Viruses

Viruses, in general, are known to activate NK cells (64, 65) and OV are no exception. One of the first reports to support the anti-tumor activation of NK cells in response to OV therapy was reported by Diaz et al. in which depletion experiments were performed to demonstrate that B16 melanoma tumor regression was achieved in a CD8^+^ T and NK cell-dependent manner following vesicular stomatitis virus (VSV) intratumoral (i.t.) injection (66). Supporting these findings, oncolytic Reovirus treatment of prostate cancer produced an anti-tumor CD8^+^ T cell response along with prominent NK cell infiltration (67, 68). Miller et al. also observed that i.t. therapy with oncolytic herpes simplex virus (HSV) for B16 melanoma was abrogated in syngeneic models lacking NK and T cell subsets (69). In mechanistic studies with oncolytic new castle disease virus (NDV), Jarahian et al. demonstrated enhanced NK cytotoxicity against human tumor cell lines infected with NDV. Further, soluble receptor binding and blocking assays suggest that NKp44 and NKp46 recognition of viral ligand hemagglutinin-neuraminidase on NDV infected tumor cells mediated NK anti-tumor activity (70). We have demonstrated that oncolytic ORF virus (ORFV) has a profound effect on NK cells following i.v. delivery and that this NK cell activation is the main mechanism by which ORFV exerts its anti-tumor effect (71). It is very likely that stimulation of NK cells play an important role in the therapeutic effect of many OV, not only by enhancing NK cell-mediated killing of tumor target cells but also by triggering a robust, T cell-mediated, anti-tumor immune response (72).

Given that surgery suppresses NK cell activity and OV activate NK cells, we explored the ability of preoperative OV to prevent postoperative NK cell suppression, and in turn prevent the development of postoperative metastases. Using our established murine model of surgical stress, we demonstrated that perioperative administration of novel oncolytic ORF and vaccinia viruses can reverse NK cell suppression following surgery and this correlates with a reduction in the postoperative formation of metastases (3). Similar effects were observed in 4T1-tumor bearing surgically stressed mice treated with perioperative OV. When NK cells were depleted, the effect was no longer present, suggesting that suppression of tumor metastases in a surgical stress model is mainly mediated through OV activation of NK cells and subsequent NK cell-mediated tumor lysis (3).

We demonstrated a similar effect with the novel oncolytic rhabdovirus, Maraba (MG1) and used this model to explore the mechanism of NK cell activation further. MG1 is a double mutant rhabdovirus with deletion in the G and M proteins (73). It is a clinical candidate OV that is scheduled to begin a Phase I clinical trial in 2014. MG1 infection in immune competent mice resulted in an immediate (24 h) and intense activation of NK cells, as evidenced by significantly increased NK cell cytotoxicity and cytokine secretion. Moreover, preoperative i.v. administration of MG1 overcame surgery-induced NK suppression and attenuated the development of postoperative metastases in the B16lacZ model of implanted lung metastases, as well as in the breast 4T1 model of spontaneous lung metastases (74).

**Figure 1:**
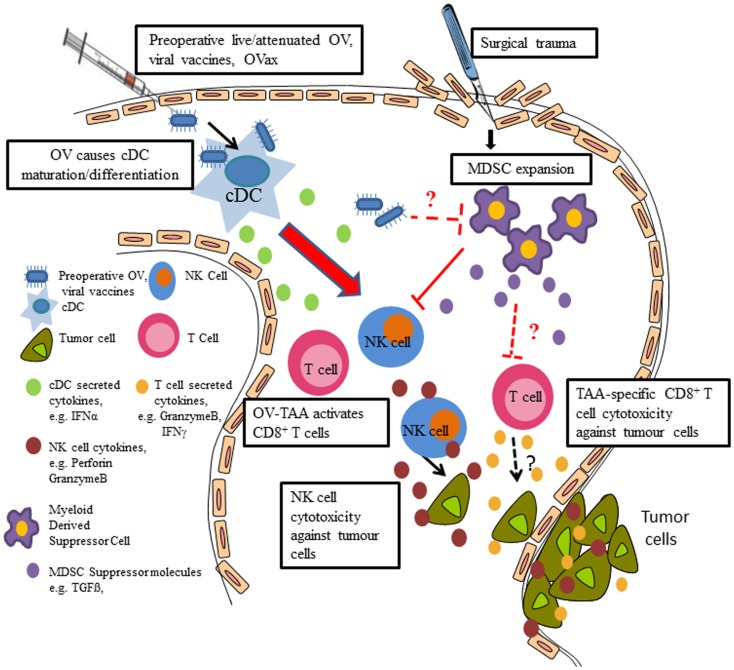
**Preoperative delivery of live/attenuated OV, viral vaccines, and oncolytic vaccines enhances innate and adaptive immune cell function to reduce postoperative metastatic disease**. Preoperative administration of the live or attenuated OV results in NK cell activation via cDC; preoperative delivery of viral vaccines results in IFNα production (likely through cDC), which results in NK cell activation, thereby preventing surgery-induced dysfunction and removal of tumor cell emboli and micrometastases in the postoperative period. Postoperative MDSC expansion contributes to NK cell dysfunction and OV may reverse the suppressive effects of MDSC. Preoperative oncolytic vaccines may activate tumor antigen-specific CD8^+^ T cells and reduce tumor burden and increase survival.

Mechanistically, we demonstrated that MG1 activates NK cells through conventional DC (cDC) (Figure 1). Using an *ex vivo* NK:DC co-culture system, we showed lack of NK infection, activation, and cytotoxicity in the absence of cDC. Further, in cDC ablated mice (CD11c-Diphtheria Toxin Receptor Transgenic mice), NK cell cytotoxicity was significantly reduced following MG1 administration (74). While we demonstrated that MG1 does not directly infect or activate NK cells, this is not the case for other OV. For instance, vaccinia virus has been shown to interact directly with NK cells through Toll-like-receptor-(TLR)-2 (75).

As the interplay between OV and immune cells in the perioperative period is critically important for the eradication of tumors, we further explored these interactions in our preclinical models of tumor and surgical stress. In both B16 melanoma and 4T1 breast tumor models, we observed postoperative expansion of myeloid-derived suppressor cells (MDSC) (3), which are known regulatory cells that have been shown to expand following various pathologies to suppress innate and adaptive immunity (76–80). The role of MDSC on surgery-induced dysfunction of NK cells and antigen-specific T cells and its potential interaction with OV is part of ongoing research in our lab (Figure 1).

## Preclinical Evidence of TAA-Specific T Cell Activation with Perioperative Oncolytic Vaccine

Oncolytic vaccines (OVax) are OV that express TAA that can direct the host immune response toward the TAA while simultaneously performing viral oncolysis and creating an inflammatory tumor microenvironment (81, 82). Dr. Brian Lichty has pioneered this prime-boost OVax platform and demonstrated remarkable efficacy in the B16 model (82–86). B16 cells express the TAA, dopachrome tautomerase (DCT), which is a protein involved in melanogenesis and is present in normal melanocytes and melanoma. As previous studies have demonstrated, Ad-DCT is able to prime a DCT specific T cell immune response and protect mice from a B16 tumor challenge or tumor re-growth (87, 88), but has limited efficacy in a therapeutic model of lung metastases (89). Dr. Lichty’s group engineered MG1, to express DCT upon productive infection and used these two viruses in a prime-boost strategy in tumor-bearing animals. They found that when Ad-DCT was allowed to prime an immune response, followed 9 days later by an MG1-hDCT boost, the results were remarkable, leading to a significant reduction in lung metastases with durable cures in >20% of mice, something not seen when MG1 expressing an irrelevant transgene (MG1-GFP, green-fluorescent protein) was used. Strikingly, ~27% of CD8^+^ T cells were directed against DCT. Selective depletion of cytotoxic T lymphocytes (CTL) at the time of the boost abrogates the therapeutic efficacy, underscoring their central role. In the near and longer term, we will focus on using OVax, such as MG1-DCT in preclinical mouse tumor models of surgical stress to perioperative boost adaptive immune functions.

## Clinical Experience with Perioperative OV in Cancer Surgery Patients

The compelling preclinical and clinical data with oncolytic vaccinia virus, in particular the evidence that it can stimulate a potent anti-tumor immune response (90) led us to hypothesize that perioperative treatment with this OV could improve recurrence-free survival following surgical resection. We designed a single center Phase II clinical trial where patients with metastatic colorectal tumors within the liver were treated with a single i.v. dose of oncolytic vaccinia virus prior to surgical resection (91). This trial explored the mechanisms of action of oncolytic vaccinia virus through a series of correlative blood and tissue studies collected from patients pre- and post-OV treatment and surgery. In this study, we confirmed that NK cell cytotoxicity improved in the setting of pre-operative oncolytic vaccinia virus compared to baseline control blood (3). Further, we detected genome copies of vaccinia virus in the tumors of patients following resection (unpublished data), which suggests that viral targeting of the tumor by i.v. injection may elicit an immune response in the tumor. These results demonstrated for the first time that oncolytic vaccinia virus markedly increases NK activity in cancer surgery patients.

In the same patient population of CRC, systemic delivery of oncolytic reovirus prior to planned surgical resection of liver metastases was undertaken by researchers in the UK (92) In this “window of opportunity” trial of 10 patients, Adair et al. was able to recover live reovirus from the blood cells, but not from plasma removed from these patients. In addition, reovirus protein was identified preferentially in resected tumor tissue, but not in normal liver tissue. Their results suggest that immune cells in the blood may protect virus from neutralizing antibodies, thus providing targeted delivery of OV to tumors. Importantly, preoperative treatment with oncolytic reovirus was well tolerated, with the most common side effects being flu-like symptoms and no reported grade 3 or 4 toxicities in any patients (92). In a study of perioperative oncolytic HSV delivery, virus was injected intratumorally pre- and post-surgical resection into patients with recurrent glioblastoma multiforme (93). Evidence of immune cell infiltration and viral replication in the resected tumors was reported by the authors. Notably, no patients developed HSV related encephalitis or required antiviral treatment (93). In a series of clinical trials using NDV-modified autologous tumor cell vaccine (NDV-ATV) for treatment of colorectal, renal cell, and glioblastoma cancer patients, researchers detected a significantly improved survival advantage compared to unvaccinated and historical controls. However, NDV-ATV was mostly administered postoperatively and not preoperatively to prevent surgery-induced immunosuppression, which might further improve upon the survival advantage. Similar to the above studies, NDV-ATV was well tolerated, with the most common side effects being mild-fever/headache and no associated autoimmunity (70, 94–99). These reports demonstrate the feasibility of perioperative OV administration into cancer surgery patients.

## The Importance of Timing for OV Administration in the Perioperative Period

While the postoperative period provides a window of opportunity for cancer cells to metastasize and grow, it also provides a window of opportunity to intervene, by strengthening the immune system and reducing recurrence of cancer following surgery in cancer patients. In our preclinical perioperative vaccine studies, we hypothesized that neoadjuvant delivery of vaccine immediately prior to surgery will allow for maximal NK cell stimulation to counteract surgery-induced NK cell suppression (100, 101). Indeed, we observed that influenza vaccine administered on the same day, immediately prior to surgery, reduced metastases most effectively. The results from NK cells isolated from cancer surgery patients also confirm that the timing of influenza administration is critical for its effect. In four out of four cancer surgery patients, NK cells isolated prior to surgical resection demonstrated enhanced cytotoxicity and IFNγ secretion following *ex vivo* pulsing with influenza vaccine, while in only one of these patients was similar activation demonstrated in NK cells isolated 1 day following surgery, suggesting that surgery-induced NK cell dysfunction can be prevented but not reversed by influenza. In humans receiving a flu shot as part of a vaccination campaign, NK cell activation peaked at 1–2 days following immunization (101). Based on this, it appears that a cancer vaccination strategy is probably best delivered the day before cancer surgery, in order to allow sufficient time to maximally activate NK cells prior to surgical stress.

Equally important for a replicating virus is the growth advantage that the postoperative state may provide, increasing oncolysis, viral replication, and spreading. Surgical stress results in a surge of VEGF with resulting angiogenesis to facilitate wound healing (21). Kottke et al. (102, 103) have previously demonstrated that a VEGF surge improved viral replication, viral cell lysis, and an innate immune mediated attack, in particular by NK cells, by allowing tumor-associated endothelial cells to transiently support viral replication during the VEGF surge. The sequential combination of oncolytic vaccinia virus and the small molecule B-raf and VEGF inhibitor, sorafenib, has also demonstrated efficacy in preclinical models and a few patients (104), further supporting the concept that OV and VEGF may act synergistically if the timing of viral administration is considered.

## Barriers to Perioperative OV Therapy and Strategies to Overcome Them

While these data are exciting, the perioperative use of OV is in preclinical and early stages of clinical investigation. In the design of our preoperative OV trial, we were confronted with multiple concerns associated with the use of a live virus immediately prior to surgery in cancer patients. In particular, concerns were raised about the potential for an overwhelming postoperative systemic inflammatory response, the risk of spread to members of the operating room team, and risk of meningitis with epidural analgesia. These safety concerns present real barriers to the development of perioperative OV. In their recent publication, Adair et al. demonstrated the feasibility and safety of perioperative live reovirus infusion prior to surgery in CRC patients. However, OV infusion was administered 6–28 days prior to surgery and not immediately before surgery. Further, three patients received fewer than their planned five doses of reovirus. In one patient, this was due to a decline in white blood cell count, while the remaining two patients opted to not receive their last doses of OV prior to surgery because of their own concerns that flu-like symptoms might interfere with the planned surgery, highlighting a strongly held belief that remains a theoretic barrier to immediate preoperative delivery of a replicating virus (92).

Given these very real concerns surrounding live perioperative delivery of OV, we subsequently focused on generating non-replicating MG1 viruses to characterize their ability to activate NK cells and attenuate metastases in a model of experimental (B16) and spontaneous (4T1) metastases following surgical stress. To accomplish this, we constructed a replication incompetent MG1 – MG1-Gless-eGFP, that is only capable of one infectious life cycle, thus offering a safe *in vivo* profile. Next, we compared these variations of MG1: (1) live MG1-productive infection and replication; (2) a G-less version (MG1-Gless) – capable of a single-replication cycle of virus; (3) MG1 exposed to ultraviolet (UV) for 2 min to 2 h – replication incompetent confirmed by plaque assay. MG1, MG1-Gless, and MG1-UV^2 min^ exhibited significantly higher NK cell function compared to PBS control, and they effectively attenuated *in vivo* B16lacZ lung metastases to near identical levels at high viral doses (1 × 10^8^ PFU). However, at all lower doses studied (1 × 10^5–7^ PFU), live MG1 demonstrated better efficacy than attenuated MG1. Furthermore, we characterized this panel of MG1 viruses in terms of virus morphological structure and cell associated interaction via Electron Microscopy, qRT-PCR, and western blot and found that MG1-UV^2 min^ remains an intact virus particle (virus proteins, genetic materials) with cell-associated interactions, corresponding to the highest NK cell activation and least lung metastases, among MG1-UV viruses. Importantly, we demonstrated that preoperative i.v. administration of equivalent high doses (1 × 10^8^ PFU) of live and attenuated MG1 (MG1-Gless or MG1-UV^2 min^) overcame surgery-induced NK cell suppression and reduced the development of postoperative metastases in the B16lacZ implanted lung metastases, as well as in the breast 4T1 model of spontaneous lung metastases. Taken together, these results suggest that the intact viral particle and cellular recognition, along with viral proteins and genomic RNA are essential for NK cell-mediated anti-tumor responses. Non-replicating forms of MG1, including MG1-UV^2 min^, are novel cancer therapies that can be safely used in the immediate preoperative period to prevent the formation of metastatic disease (74).

Parallel to our perioperative attenuated OV studies, we assessed a wide range of potential agents to provide perioperative non-specific immunostimulation including TLR ligands and inactivated vaccines against infectious disease. Firstly, we assessed a panel of routinely used immunizations, including vaccines against influenza, meningitis, measles/mumps/rubella, diphtheria/tetanus/pertussis/polio, pneumonia, and influenza for their ability to activate (CD69 expression) and enhance NK cell function (cytotoxicity and IFNγ secretion). When directly compared, influenza was the most potent NK cell activator among the prophylactic vaccines, although, not unexpectedly, inoculating mice with live replicating viruses (such as vaccinia virus) induced higher levels of NK cell cytotoxicity. Using our mouse models of experimental (B16 melanoma) and spontaneous (4T1) metastases and surgical stress, we subsequently demonstrated that preoperative delivery of a single dose of influenza resulted in a dramatic reduction in lung metastases (101). In order to confirm that NK cells play a mediating role in preventing postoperative metastases following influenza treatment, we pharmacologically depleted NK cells and observed a complete abrogation of the therapeutic effect of influenza vaccination. Furthermore, we discovered that IFNα had the most dramatic increase following influenza vaccination after assessing a panel of serum cytokines following influenza administration. We also observed that low-dose preoperative IFNα was able to rescue surgery-induced NK cell dysfunction and metastases to the same degree as influenza vaccination. The central role for IFNα was underscored by demonstrating that influenza vaccination was not able to increase postoperative NK cell activity or attenuate postoperative metastases in IFNα receptor-deficient mice. In PBMC isolated from human donors, Type I IFN blocking antibody prevented influenza from activating NK cells (101). While our study did not explore the role of DC in the production of IFNα following influenza vaccination, it is very likely that they represent the primary source, resulting in secondary NK cell stimulation (see Figure 1).

## Clinical Implications and Future Directions

Surgical resection is the mainstay of therapy for patients with localized solid malignancies. Even with complete resection, many patients develop a metastatic recurrence and ultimately die of their disease. The immediate postoperative period provides an ideal environment for the formation of cancer metastases. Despite this, it remains a therapeutic window that is largely ignored. There are currently no standard perioperative anti-cancer therapies aimed at preventing postoperative metastases. We have demonstrated in preclinical models that perioperative OV therapy can activate both the innate and adaptive immune responses and attenuate metastatic disease. Early clinical trials confirm the feasibility of this strategy but these therapies must be rigorously characterized for safety and efficacy and then translated into thoughtfully designed clinical trials. This research supports the concept that neoadjuvant (preoperative) OV treatments can reverse postoperative immune dysfunction, while directly infecting and killing tumor cells and creating a favorable immune microenvironment. This treatment strategy has the potential to impact countless cancer patients who undergo surgical resection of their solid tumor every year.

## Conflict of Interest Statement

The authors declare that the research was conducted in the absence of any commercial or financial relationships that could be construed as a potential conflict of interest.
